# Unveiling the invisible: Is there a role of CMR in biopsy‐negative graft dysfunction post‐heart transplantation?

**DOI:** 10.1002/ehf2.14994

**Published:** 2024-07-23

**Authors:** Justyna M. Sokolska, Robert Manka

**Affiliations:** ^1^ Department of Cardiovascular Imaging, Faculty of Medicine, Institute of Heart Diseases Wroclaw Medical University Wroclaw Poland; ^2^ Institute of Heart Diseases Wroclaw University Hospital Wroclaw Poland; ^3^ Department of Cardiology, University Heart Center University Hospital Zurich Zurich Switzerland; ^4^ Institute of Diagnostic and Interventional Radiology University Hospital Zurich Zurich Switzerland; ^5^ Institute for Biomedical Engineering University and ETH Zurich Switzerland

Cardiac magnetic resonance (CMR) is widely used in diagnosing cardiomyopathies, heart failure of unknown aetiology, and ischaemic heart diseases.^1^, ^2^ In recent years, CMR has become a valuable tool for determining the underlying cause in acute conditions such as myocardial infarction with non‐obstructive arteries (MINOCA) and myocarditis.^3^, ^4^, ^5^, ^6^, ^7^ In most myocarditis cases, the diagnosis can be confirmed based on clinical history and evidence of myocardial inflammation on CMR. Using the original Lake Louise criteria from 2009, which were developed to standardize the diagnosis of myocarditis based on CMR images, this technique demonstrated good specificity and sensitivity for detecting myocardial oedema.^8^ In 2018, these criteria were revised to include a new CMR sequence featuring paramagnetic mapping.^9^ It is recommended to perform CMR within the first 10–14 days from symptoms onset to increase the chance of oedema visualization.^10^ Tissue inflammation can be visualized on CMR using T2‐based images, such as Short Tau Inversion Recovery (STIR) or modern T2‐mapping. Additionally, with new CMR sequences such as T1‐mapping, extracellular volume calculation, and T2*‐mapping, CMR can monitor disease progression and treatment response in patients with conditions like amyloidosis, Fabry disease, or haemochromatosis.^11^ Recent studies have investigated changes in CMR parameters in heart failure patients with iron deficiency undergoing iron supplementation.^12^

After heart transplantation, it is crucial to closely monitor patients for signs of early cellular and humoral rejection.^13^ The gold standard to monitor cellular rejection is the endomyocardial biopsy, which is a planned procedure and is performed about 12 times in the first year. Based on the current histopathologic findings, where bioptomes are assessed according to the 2005 International Society of Heart and Lung Transplantation (ISHLT) heart biopsy grading scale, the immunotherapy is adjusted for each patient.^14^ However, due to the invasiveness and potential complications of endomyocardial biopsy, there has been significant focus, especially during the COVID‐19 pandemic, on finding alternative, non‐invasive methods to assess rejection in post‐transplant patients. It is not surprising that CMR was one of the first techniques considered, due to its unique tissue characterization capabilities and only few contraindication.^15^, ^16^ One of the most significant studies in this field was a single‐centre randomized controlled trial by Anthony et al., which demonstrated that a CMR‐based multiparametric mapping approach (combining T1‐ and T2‐mapping) during the first year after orthotopic heart transplantation is highly reproducible and reliable for detecting acute cardiac allograft rejection, making it a feasible alternative to endomyocardial biopsy‐based surveillance.^17^

A much more complex topic is biopsy‐negative graft dysfunction (GD) after heart transplantation (HTx). The pathophysiology, appropriate diagnostic criteria, and optimal treatment for these patients remain poorly understood.^18^ The varying definitions of biopsy‐negative GD lead to heterogeneous study populations. It is typically diagnosed when cardiac dysfunction is observed, defined by a decline in left ventricular ejection fraction (LVEF) to ≤45%, despite normal histological findings and no other causes of cardiac allograft damage. Importantly, it has been shown that patients with haemodynamic compromise and a low ISHLT score have worse outcomes than those with a high score (indicating cellular rejection). It is believed that humoral rejection, involving immunologic mechanisms other than lymphocytic infiltration, is the primary cause of biopsy‐negative GD, occurring in 10 to 20% of heart‐transplanted patients.^18^

In this context, Anand et al. report in the current issue of *ESC Heart Failure* on the use of CMR in diagnosis biopsy‐negative GD in post–Htx patients.^19^ As Htx remains a crucial treatment option for patients with end‐stage heart failure, the authors are to be commended for their efforts to use modern cardiac imaging to better understand the rare and complex complication of biopsy‐negative GD. Researchers from the Mayo Clinic in the USA defined biopsy‐negative GD as a decrease in LVEF by more than 5% and an LVEF below 50%, in the absence of biopsy‐proven rejection or allograft vasculopathy. This definition was met by 34 Htx recipients who underwent CMR between 2007 and 2020. The presented retrospective study had three aims: (1) to examine CMR findings in heart transplant recipients with biopsy‐negative GD, (2) to evaluate outcomes based on the presence of LGE in CMR, and (3) to identify potential missed rejection cases in endomyocardial biopsy using CMR. The primary composite outcome consisted of all‐cause mortality, re‐transplantation, or persistent LVEF <50%. Regarding the first study aim, the authors shown that almost half of patients had presence of late gadolinium enhancement (LGE), all of non‐ischaemic pattern. Interestingly, patients with LGE experienced GD later (4 vs. 1 year after Htx) were more often symptomatic (88% vs. 56%) and had higher pulmonary capillary wedge pressure (19 ± 7 vs. 13 ± 3 mmHg, all *P* < 0.05) compared with those without any LGE. Importantly, those differences had no significant impact on patients outcomes, including the primary composite outcome and improvement of LVEF during the median follow up of 3.8 years. These findings highlight the complex pathophysiology of biopsy‐negative GD, where inflammation accounts for about 50% of cases, while neurohormonal and immunomodulatory mechanisms are significant in the remaining patients. Another important takeaway is that treatment decisions should not rely solely on CMR findings, as LGE is absent in over half of patients with biopsy‐negative GD.

However, there are some issues to note regarding the study by Anand et al. The main limitation of the study is that no parametric mapping techniques were tested, as the patients were retrospectively recruited from quite a long time period that covered 13 years, starting in 2007. It would have been very valuable to assess T1‐ and T2‐mapping in that population of patients with biopsy‐negative GD, as multiparametric approaches are already very promising in acute cardiac allograft rejection.^15^, ^17^ Another limitation is the retrospective nature and small sample size due to the rare occurrence of biopsy‐negative GD. Therefore, it would be beneficial to conduct similar studies prospectively with a multicentre design and to include CMR findings in prospective multinational registries for post‐HTx patients. Finally, the definition of biopsy‐negative GD, based on a mere 5% decrease in LVEF resulting in an LVEF below 50%, may be unreliable in borderline cases due to inter‐ and intraobserver variability in LVEF measurement. This variability could lead to inconsistent diagnoses and underscores the need for more precise criteria.

In conclusion, CMR imaging is emerging as a valuable diagnostic tool for post‐HTx patients who require close monitoring for signs of organ rejection. Its unique tissue characterization ability, non‐invasive nature, and increasing availability make it a promising alternative. Growing evidence supports its effectiveness in detecting acute cellular rejection, offering a feasible option compared to the gold standard of endomyocardial biopsy. In the rare condition of biopsy‐negative GD, CMR shows promise as a complementary diagnostic tool for better understanding underlying pathomechanisms. However, this field is still in its early stages and requires further exploration.

## References

[1] Kan A , Fang Q , Li S , Liu W , Tao X , Huang K , *et al*. The potential predictive value of cardiac mechanics for left ventricular reverse remodelling in dilated cardiomyopathy. ESC Hear Fail 2023;10:3340‐3351. 10.1002/ehf2.14529 PMC10682859 37697922

[2] Bai L , Wang Y , Chen F , Peng Y . Hypertrophic cardiomyopathy with heart failure and ST‐segment elevation of the lateral wall. ESC Hear Fail 2023;10:705‐708. 10.1002/ehf2.14185 PMC9871721 36178133

[3] Berg J , Kottwitz J , Baltensperger N , Kissel CK , Lovrinovic M , Mehra T . Cardiac magnetic resonance imaging in myocarditis reveals persistent disease activity despite normalization of cardiac enzymes and inflammatory parameters at 3‐month follow‐up. Circ Heart Fail 2017;10:e004262. 29158437 10.1161/CIRCHEARTFAILURE.117.004262

[4] Castrichini M , Porcari A , Baggio C , Gagno G , Maione D , Barbati G , *et al*. Sex differences in natural history of cardiovascular magnetic resonance‐and biopsy‐proven lymphocytic myocarditis. ESC Hear Fail 2022;9:4010‐4019. 10.1002/ehf2.14102 PMC9773744 36000547

[5] Patriki D , Gresser E , Manka R , Emmert MY , Lüscher TF , Heidecker B . Approximation of the incidence of myocarditis by systematic screening with cardiac magnetic resonance imaging. JACC Heart Fail 2018;6:573‐579. 29885953 10.1016/j.jchf.2018.03.002

[6] Usui E , Nagaoka E , Ikeda H , Ohmori M , Tao S , Yonetsu T , *et al*. Fulminant myocarditis with COVID‐19 infection having normal C‐reactive protein and serial magnetic resonance follow‐up. ESC Hear Fail 2023;10:1426‐1430. 10.1002/ehf2.14228 PMC10053182 36401586

[7] Amelotti N , Mapelli M , Guglielmo M , Pires MIFB , Campodonico J , Majocchi B , *et al*. What's behind your eosinophilic myocarditis? A case of Churg–Strauss syndrome diagnosed during acute heart failure. ESC Hear Fail 2023;10:709‐715. 10.1002/ehf2.14172 PMC9871676 36259268

[8] Friedrich MG , Sechtem U , Schulz‐Menger J , Holmvang G , Alakija P , *et al*. Cardiovascular magnetic resonance in myocarditis: A JACC white paper. J Am Coll Cardiol 2009;53:1475‐1487. 19389557 10.1016/j.jacc.2009.02.007 PMC2743893

[9] Ferreira VM , Schulz‐Menger J , Holmvang G , Kramer CM , Carbone I , Sechtem U , *et al*. Cardiovascular magnetic resonance in nonischemic myocardial inflammation: Expert recommendations. J Am Coll Cardiol 2018;72:3158‐3176. 30545455 10.1016/j.jacc.2018.09.072

[10] Mileva N , Paolisso P , Gallinoro E , Fabbricatore D , Munhoz D , Bergamaschi L , *et al*. Diagnostic and prognostic role of cardiac magnetic resonance in MINOCA: Systematic review and meta‐analysis. JACC Cardiovasc Imaging 2023;16:376‐389. 36889851 10.1016/j.jcmg.2022.12.029

[11] Wu YK , Tsai CH , Su MY , Chao CC , Cheng MF , Shun CT , *et al*. Reverse cardiac remodelling and dysfunction in A97S transthyretin cardiac amyloidosis after tafamidis treatment. ESC Hear Fail 2022;9:4335‐4339. 10.1002/ehf2.14165 PMC9773766 36128649

[12] Gertler C , Jauert N , Freyhardt P , Valentova M , Aland SC , Walter‐Rittel TC , *et al*. Magnetic resonance imaging of organ iron before and after correction of iron deficiency in patients with heart failure. ESC Hear Fail 2023;10:1847‐1859. 10.1002/ehf2.14329 PMC10192268 36907649

[13] Scheiber D , Zweck E , Albermann S , Jelenik T , Spieker M , Bönner F , *et al*. Human myocardial mitochondrial oxidative capacity is impaired in mild acute heart transplant rejection. ESC Hear Fail 2021;8:4674‐4684. 10.1002/ehf2.13607 PMC8712779 34490749

[14] Stewart S , Winters GL , Fishbein MC , Tazelaar HD , Kobashigawa J , Abrams J , *et al*. Revision of the 1990 working formulation for the standardization of nomenclature in the diagnosis of heart rejection. J Hear Lung Transplant 2005;24:1710‐1720. 10.1016/j.healun.2005.03.019 16297770

[15] Dolan RS , Rahsepar AA , Blaisdell J , Suwa K , Ghafourian K , Wilcox JE , *et al*. Multiparametric cardiac magnetic resonance imaging can detect acute cardiac allograft rejection after heart transplantation. JACC Cardiovasc Imaging 2019;12:1632‐1641. 30878427 10.1016/j.jcmg.2019.01.026 PMC6995349

[16] Akhyari P , Immohr MB , Bönner F , Reinecke P , Lichtenberg A , Boeken U . Transplantation of hearts from SARS‐CoV‐2 positive donors. ESC Hear Fail 2023;10:2698‐2701. 10.1002/ehf2.14389 PMC10375114 37088468

[17] Anthony C , Imran M , Pouliopoulos J , Emmanuel S , Iliff J , Liu Z , *et al*. Cardiovascular magnetic resonance for rejection surveillance after cardiac transplantation. Circulation 2022;145:1811‐1824. 35621277 10.1161/CIRCULATIONAHA.121.057006

[18] Fishbein MC , Kobashigawa J . Biopsy‐negative cardiac transplant rejection: Etiology, diagnosis, and therapy. Curr Opin Cardiol 2004;19:166‐169. 15075746 10.1097/00001573-200403000-00018

[19] Anand S , Alnsasra H , LeMond LM , Shrivastava S , Asleh R , Rosenbaum A , *et al*. Cardiac magnetic resonance imaging in heart transplant recipients with biopsy‐negative graft dysfunction. ESC Hear Fail 2024;11:1594‐1601. 10.1002/ehf2.14681 PMC11098666 38379022

